# Raising the Stakes for Online Learning: Monetary Incentives Increase Performance in a Computer-Based Learning Task Under Certain Conditions

**DOI:** 10.3389/fpsyg.2022.780301

**Published:** 2022-05-05

**Authors:** Jessica F. Schwab, Leah H. Somerville

**Affiliations:** Department of Psychology, Harvard University, Cambridge, MA, United States

**Keywords:** goal-directed, cognition, incentives, stakes, online learning

## Abstract

To what extent can external incentives influence students’ effort and learning in online course contexts? While cognitive science research has found that monetary incentives can increase goal-directed cognitive effort in certain laboratory tasks, attempts to use monetary incentives to increase students’ academic performance in naturalistic settings has shown mixed results. In two experiments, we tested the influence of a monetary incentive (compared to no external incentive) on immediate and delayed tests of computer-based educational performance (i.e., learning from educational videos). In Experiment 1, participants were assigned to (1) receive monetary incentives for correct quiz responses, or (2) receive no additional incentive for correct responses other than finding out their score, and we found no significant difference in total score across groups (on either immediate or delayed tests of learning). In Experiment 2, we used a within-subjects design to test whether participants performed better when they were provided monetary incentives for correct responses on quiz questions (compared to no external incentive). Here, participants performed significantly better on incentivized quiz questions (on both immediate and delayed tests of learning). Thus, monetary incentives may increase performance in online learning tasks when participants can anchor the “stakes” of an incentive compared to no external incentive. These findings highlight potential benefits of external incentives for promoting effort and learning in online contexts, although further research is needed to determine the most useful educationally-relevant extrinsic incentives, as well as potential negative effects of incentives on long-term intrinsic motivation.

## Introduction

Improving students’ engagement and learning in online course environments is of ever-growing importance, particularly given the sudden increased use of remote learning and availability of online courses. In a computer-based learning context devoid of peer interaction or the watchful eye of a professor, frequent occurrences of mind-wandering are known to take place (Szpunar et al., 2013; Hollis and Was, 2016). How can educators and policymakers promote students’ effort and learning in these contexts, given the increased likelihood of distraction? Cognitive science research suggests that monetary incentives can increase goal-directed cognition in the laboratory, including attentional effort and learning (e.g., Hübner and Schlösser, 2010; Davidow et al., 2018). However, attempts to use monetary incentives to increase students’ academic performance in naturalistic settings have shown mixed results (O’Neil et al., 1995; Fryer, 2011; Levitt et al., 2016). In order to reconcile these conflicting findings regarding the use of external incentives and goal-directed learning behaviors, the present study aims to directly examine whether monetary incentives can increase performance in a laboratory-based online learning context.

Extensive research suggests that individuals utilize cues of value in carrying out goal-directed behavior (e.g., Braver et al., 2014; Davidow et al., 2018). For example, in a word memorization task where certain words had higher or lower “value” (i.e., were worth more or less points), adults over the age of 18 selectively directed their cognitive resources toward remembering higher-value words (e.g., Castel et al., 2011). Monetary incentives have been shown to have a particularly strong effect on effortful cognition, including selective attention (Libera and Chelazzi, 2006) and cognitive control (i.e., the process of inhibiting automatic responses in service of a goal) (Locke and Braver, 2008; Chiew and Braver, 2014). Moreover, neuroimaging research has revealed that monetary incentives enhance processing in brain regions that mediate control of attention (Small et al., 2005), working memory (Krawczyk et al., 2007), and cognitive control (Locke and Braver, 2008).

Although monetary incentives seem to increase individuals’ motivation toward accomplishing effortful cognitive tasks in the laboratory, there have been mixed results in the field of education regarding the use of monetary incentives for increasing academic performance. In a series of large-scale field experiments with 2nd-, 4th-, 7th-, and 9th-grade students from three typically low-performing urban school districts – Chicago, Dallas, and New York City – researchers found that monetary incentives had little, if any, significant influence on academic performance (either paying students to increase their reading, paying for performance on interim assessments, or paying for classroom grades) (Fryer, 2011). In another study, O’Neil et al. (1995) found that 8th graders performed significantly better on a standardized mathematics test when given monetary incentives ($1 per question correct) compared to no incentive control conditions, but 12th graders performed no better when given monetary incentives. Even when they were offered $10 per test item correct on a low-stakes standardized test, 12th-grade students did no better compared to a no-money control condition (O’Neil et al., 2005).

Other researchers have examined the influence of paying students for individual *improvement*, again with mixed results. Baumert and Demmrich (2001) offered 9th-grade German students ten Deutsch Marks for solving more items than would be expected on the basis of their previous mathematics grade, and they found no significant influence of monetary incentives on performance. On the other hand, Levitt et al. (2016) performed a series of field experiments with over 6,000 students (2nd-8th graders and 10th graders) from three low-performing schools near Chicago and revealed a significant effect of monetary incentives on improvement in standardized test scores, particularly when incentives were framed as a “loss.” Specifically, students were given $10 or $20 before the test and were told they could only keep the money if they showed improvement in test scores (compared to a previous test). However, their results were mixed for monetary incentives framed as a “gain,” where students were told they would receive an additional $10 or $20 for test improvement (Levitt et al., 2016).

One possible explanation for these mixed results is that offering incentives *immediately* following a testing instance (as opposed to a later time point or at the end of the semester) are more likely to shape student effort and performance (Levitt et al., 2016). Braun et al. (2011) found that offering 12^th^-grade students $15 per correct response (for two randomly chosen questions) immediately after an exam improved their performance on a standardized reading assessment, as well as their self-reported engagement and effort. Moreover, Levitt et al. (2016) found an effect of immediate monetary incentives on improvement in student test performance, but they no longer saw a significant effect of incentives on improvement in test performance when the incentive was delayed (one month following the exam) (Levitt et al., 2016).

Research with college-aged students, however, has revealed benefits of delayed incentives on academic performance, although this effect seems highly context-dependent. One study found that offering merit-based scholarships (i.e., cash awards) for students meeting a target GPA improved women’s, but not men’s, semester grades (Angrist et al., 2009). Another field experiment offered first-year university students in Amsterdam monetary incentives for passing all of their required first-year exams (Leuven et al., 2010). These researchers found a positive effect of incentives on achievement (in terms of higher pass rates and more credit points) for high-ability students, but a negative effect on achievement for low-ability students (Leuven et al., 2010).

Thus, although monetary incentives clearly influence goal-based cognitive performance in the laboratory, it is still unclear whether these types of incentives can have a significant influence on academic achievement. Given the subjective nature of “value” (e.g., Davidow et al., 2018), perhaps in an educational context, money is simply not a relevant, “valuable” incentive that successfully drives students to engage in academic tasks. It is also possible that students actually learn more in learning environments *without* any extrinsic motivators; they may acquire knowledge most effectively without being hyper-focused on performance. Therefore, the present study aims to directly test the influence of offering an immediate monetary incentive – compared to no monetary incentive – on performance in a computer-based educational context.

Importantly, it is also possible there may be negative long-term consequences to offering external incentives for academic performance. Previous research has shown that external incentives might *decrease* intrinsic motivation in the future (also known as the “undermining effect”) (see Braver et al., 2014 for a review of this literature). Lepper et al. (1973) presented early evidence for this idea by finding that children were less likely to spend time practicing drawing if they had previously received a “Good Player Award” for their drawing, compared to a control condition where they had not received this external incentive. However, the generalizability and replicability of this finding has been widely debated. One meta-analysis found that overall, incentives did not significantly undermine intrinsic motivation (Cameron and Pierce, 1994), while another found that they *did* (Deci et al., 1999), and these two theoretical camps have been in continued debate (e.g., Cameron et al., 2001; Deci et al., 2001). A more recent cognitive neuroscience study found additional evidence that providing an external monetary incentive for successful performance on a task decreased intrinsic motivation (Murayama et al., 2010). In this case, participants who had initially been paid to practice a stopwatch task (i.e., successfully stop a stopwatch at an exact time point) showed significantly decreased time engaging in the task once the monetary incentive was removed, compared to a control group of participants who had never received the incentive (Murayama et al., 2010). Given these findings, in the present study we also aimed to test whether any effects of monetary incentives on online learning performance were long-lasting, or if they decrease motivation and performance over time (i.e., result in an “undermining effect”). Therefore, we also tested participants’ performance on a follow-up online quiz approximately one week later, which measured the retention of their in-lab learning.

To our knowledge, no empirical research has directly compared the influence of monetary incentives (compared to no external incentive) on performance in an online (i.e., computer-based) learning context. If money does in fact influence goal-directed cognitive effort in an online educational context, then we expected participants to perform better in the monetary incentive condition (compared to no external incentive). Moreover, if money motivates participants to direct effort into learning, then we also expected to see a significant improvement in performance on a delayed test of learning. However, if extrinsic incentives interact negatively with intrinsic motivation, then results may reveal decreased performance over time for monetarily incentivized participants.

## Experiment 1

Experiment 1 tested the effect of monetary incentives on performance in a computer-based learning task. Participants were either assigned to a *Monetary* condition or *No Incentive* condition. All participants were brought into the lab to watch four online educational videos and answer 32 content-based quiz questions about the videos. In the Monetary condition, participants were told they would see their score at the end and receive 50 additional cents per question they answered correctly (up to $16 in bonus money). In the No Incentive condition, participants were simply told they would see their score at the end. If monetary incentives increase cognitive effort in a computer-based learning task, we expected participants to show more successful performance in the Monetary condition compared to the No Incentive condition.

### Method

#### Participants

106 healthy college-aged adults took part in Experiment 1 (30.19% male, 32.08% Caucasian, age range: 18-22, *M* = 20.38, *SD* = 1.30). Participants were recruited through the Affective Neuroscience and Development Laboratory database (advertised in local newspapers, flyers, and online forums) and through Harvard University’s SONA Study Pool. Exclusion criteria included having a history of or current psychiatric diagnosis, past or present use of psychotropic drugs, having a learning disability diagnosis, and being fluent in English by age 12. Participants recruited through the lab database were pre-screened for our exclusion criteria, but we did not pre-screen participants who signed up through the SONA system and therefore tested some participants who did not meet inclusion criteria. Thus, 36 additional participants were tested but not included due to psychiatric diagnosis and/or psychotropic drugs (*N* = 17 participants), learning disability (*N* = 2), being non-fluent in English or becoming fluent after the age of 12 (*N* = 11), incomplete reporting of medical history (*N* = 1), or a combination of criteria (*N* = 5). Note that we also ran an additional between-subjects group of 72 participants (19 excluded due to exclusion criteria) in a condition not reported here. We removed this condition from the present paper in order to provide a more direct comparison across experiments. However, results remain the same across analyses with this condition included, and these additional analyses can be found in the Supplementary Materials.

Published data on laboratory experiments examining the effect of incentives on online learning were not available to inform *a priori* power analyses. Thus, we aimed to ensure that the sample size was sufficiently powered to detect medium-sized effects. A power analysis using the pwr2 package in R suggested that 53 participants per group would be required for a medium effect size (Cohen’s *f* = 0.25) at *p* < 0.05 and 80% power.

Participants provided informed consent for the present study, and all research procedures were approved by the Committee for Use of Human Subjects at Harvard University.

#### Materials

All participants watched four educational videos developed for the TED-Ed project, TED’s education initiative: “The Unexpected Math Behind Van Gogh’s Starry Night” (St. Clair, 2014), “The Real Story Behind Archimedes’ Eureka!” (D’Angour, 2015), “Inside the Killer Whale Matriarchy” (Croft, 2018), and “How Tall Can a Tree Grow?” (Hammoudi, 2019). Videos ranged from four- to five-minutes in length, featured the same style of animated graphics, and included voice-over from the same narrator. Access to TED-Ed videos was granted per TED’s Creative Commons license.

Participants also completed eight multiple-choice content questions per video (32 questions total, with five response options per question). Video order and question order were identical across all participants, but order of multiple-choice response options was randomized across participants. An example multiple-choice question is as follows, “What was the primary purpose for why the king of Syracuse wanted to commission the largest ship ever?,” with the following response options: “To profit from the thousands of passengers who would ride the ship,” “To give as a present to Egypt’s ruler,” “To promote scientific enterprise,” “To accomplish the feat before the city of Alexandria did (similar to the “space race” of modern times),” or “Because he owed Archimedes a favor”. Total score on the online learning task was calculated as the number of questions correct out of 32. Participants’ response time to answer each question was also automatically recorded.

Participants also completed the matrix reasoning subtest of the Wechsler Abbreviated Scale of Intelligence (WASI-II) (Wechsler, 2011), and we used raw scores on this scale as a proxy for cognitive ability. Additionally, we assessed participants’ general background knowledge about the topics being portrayed in the videos. Specifically, we asked participants, “Please rank your level of knowledge about the following topics (where “1” is not at all knowledgeable and “5” is very knowledgeable)”. The topic areas were as follows: Vincent Van Gogh, turbulent flow, the story of Archimedes’ eureka moment, Archimedes’ principle, the lives of killer whales, general knowledge about marine ecosystems, how tall trees grow, and general knowledge about plant biology.

Finally, participants completed an at-home 32-question quiz consisting of eight new content questions per video, with five new multiple-choice options per question. Participants were aware they would be re-contacted for a follow-up questionnaire, but did not know they would be quizzed about the content of the videos. Question order was identical across all participants, but order of multiple-choice response options was randomized across participants. Total score on the follow-up learning assessment was again calculated as the number of questions correct out of 32, and participants’ response time to answer each question was also automatically recorded.

#### Procedure

The experiment room was designed to be similar to a standardized test-taking environment. At each in-lab session, participants arrived at a computer lab and were given a subject identification number. An average of 5.07 participants (*SD* = 3.66) took part in each session (range: 1-12). They were told to sit at an individual computer (separated from other computers by dividers), put headphones on, follow the instructions on their computer screen, and raise their hand if they had questions for the experimenter. Participants were assigned either to a *Monetary* condition or *No Incentive* condition, depending on their laboratory session (all participants in a given session were assigned to the same condition).

**FIGURE 1:**
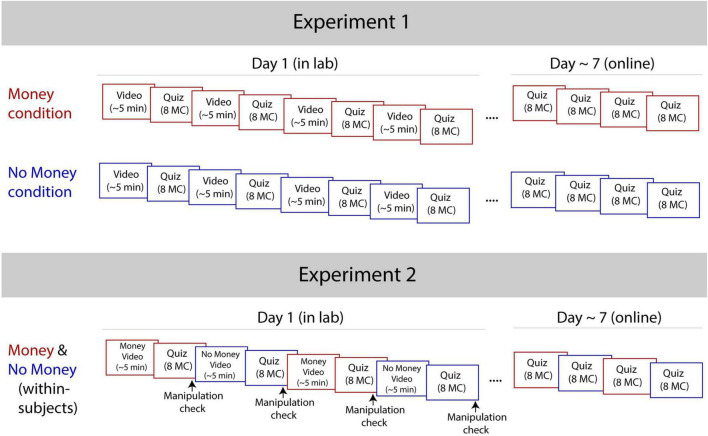
Design of Experiment 1 and Experiment 2. Across experiments, participants were shown the same videos (approximately five minutes each) and took the same multiple-choice (MC) quiz questions. Each video was immediately followed by a corresponding quiz. All participants saw their final score at the end of the lab experiment (Day 1) but received no feedback after the online follow-up (Day ∼7). In Experiment 1, participants were either assigned to the Monetary condition or No Incentive condition, but all participants watched the videos in the same order. In Experiment 2, all participants received the same instructions, where two of the videos were incentivized monetarily and the other two were not. Video order was randomized across participants for Experiment 2. In Experiment 2, after each quiz, participants also received a manipulation check (four total) to ensure they remembered the preceding video type (Monetary or No Incentive).

Participants first provided informed consent, answered demographic questions, reported their background topic knowledge, and completed the matrix reasoning WASI-II subtest (Wechsler, 2011). Next, participants received instructions for completing the in-lab learning task. All participants were told the following: “In this part of the study, you will watch four five-minute educational videos and answer questions about what you learned. You will complete a short quiz following each of the four videos.” For the Monetary condition, participants received the following additional instructions: “You will also receive bonus money based on how well you do on this online learning task. At the end of the task (32 questions total), you will see your final score and be paid 50 additional cents for each question you answered correctly (up to $16).” For the No Incentives condition, participants were told, “You will receive information about how well you did on this online learning task. At the end of the task, you will see your final score.” Participants watched four TED-Ed videos and completed eight multiple-choice content questions immediately following each video (see Figure 1). At the end of the study, participants in both the Monetary and No Incentive conditions were shown their total score. All participants received standard compensation for participating in the lab study ($15 per hour or course credit). Participants in the Monetary condition were also paid 50 additional cents for every question they answered correctly.

Approximately one week later (between 6-9 days following the in-lab experiment), participants completed an additional online “questionnaire.” This surprise follow-up quiz consisted of 32 new multiple-choice content questions based on the videos that participants watched in lab (see Figure 1). The follow-up quiz questions were identical across participants, and participants were given no additional compensation beyond standard compensation (a $10 gift card). Participants received the following instructions: “In what follows, you will be asked a series of questions about what you learned from the videos you watched during Part 1. **Please do not look up any information about these videos.** Just try your best, and answer the questions as quickly as possible. Also note that you will *not* be shown your score at the end of this task, and you will not receive any additional compensation for answering the questions correctly.” At the end of the study, participants were asked to report if they had re-watched any of the TED-Ed videos since watching them in the lab-based experiment.

#### Analytical Approach

Analyses examined the effect of incentive condition (Monetary vs. No Incentive) on total quiz score. Linear mixed-effects models were computed separately for the in-lab and online follow-up scores using the *lmer* function from the *lme4* (Bates et al., 2015) and *lmerTest* packages in R (Kuznetsova et al., 2017), with condition (Monetary vs. No Incentive) as the key predictor of interest. Video type (referring to the four TED-Ed videos) was included as a fixed effect (to account for any differences in difficulty across videos), and subject (i.e., participant) was included as a random effect (lme4 model formula: *Score* ∼ *Condition* + *Video* + (*1* | *Subject)*).

Given previous research showing possible gender-based (Angrist et al., 2009) and ability-based differences (Leuven et al., 2010) in the influence of monetary incentives on academic achievement, we also re-ran all models with gender (self-reported gender identity) and cognitive ability (as measured by the matrix reasoning subtest of the WASI-II) included as fixed effects (lme4 model formula: *Score* ∼ *Condition* + *Video* + *Gender* + *WASI* + (*1* | *Subject)*).

Additionally, we ran analyses to determine if there was a difference in average response time to quiz questions based on incentive condition (Monetary vs. No Incentive). Linear mixed-effects models were again computed separately for the in-lab and online follow-up scores, with condition (Monetary vs. No Incentive) as the key predictor of interest. Video type was included as a fixed effect, and subject (i.e., participant) was included as a random effect (lme4 model formula: *ResponseTime* ∼ *Condition* + *Video* + (*1* | *Subject)*).

#### Results and Discussion

First, we found no significant difference in raw scores on the matrix reasoning subtest between participants in the Monetary condition (*M* = 24.28, *SE* = 0.36) and No Incentive condition (*M* = 23.92, *SE* = 0.32), *t*(104) = 0.75, *p* = 0.46, *d* = 0.15. This suggests that participants did not differ across conditions on our measure of cognitive ability. Additionally, average background topic knowledge was low overall (*M* = 2.06 out of 5, *SE* = 0.07), and we found no significant difference in level of background knowledge between participants in the Monetary Condition (*M* = 2.05, *SE* = 0.09) and No Incentive Condition (*M* = 2.07, *SE* = 0.10), *t*(104) = 0.14, *p* = 0.89, *d* = 0.03.

**FIGURE 2:**
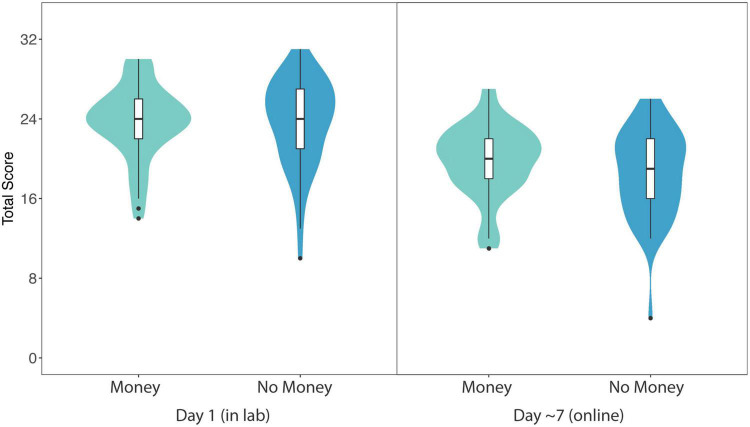
Violin plots showing probability density of total score data for Monetary and No Incentive conditions in Experiment 1 (in lab and online follow-up). Maximum score was 32 points (8 questions per video). Horizontal lines show median average score, surrounded by boxes indicating the interquartile range. Results revealed no significant effect of condition (Monetary vs. No Incentive) on total score in the lab experiment or the online follow-up.

Figure 2 displays mean total in-lab quiz scores (out of 32) for participants in the Monetary condition (*M* = 23.70, *SE* = 0.50) and No Incentive condition (*M* = 23.66, *SE* = 0.61). Our primary linear mixed-effects model revealed no significant main effect of condition on total quiz score (β < 0.01, *SE* = 0.17, 95% CI: [−0.377, 0.396], *p* = 0.96). When gender and cognitive ability were included in the model as fixed effects, the model again revealed no significant main effect of condition on total quiz score (β = 0.02, *SE* = 0.20, 95% CI: [−0.400, 0.357], *p* = 0.91) and no main effect of gender on total quiz score (β = 0.02, *SE* = 0.20, 95% CI: [−0.536, 0.289], *p* = 0.91). However, there was a significant main effect of cognitive ability on total quiz score, with higher matrix reasoning scores predicting better quiz scores (β = 0.09, *SE* = 0.04, 95% CI: [0.009, 0.163], *p* = 0.03).

To examine the influence of incentives on delayed quiz performance, we compared mean total follow-up quiz scores (out of 32) for participants in the Monetary condition (*M* = 19.84, *SE* = 0.50) and No Incentive condition (*M* = 18.56, *SE* = 0.61) one week later (see Figure 2). Note that two participants did not complete the follow-up, and we excluded scores of three participants for completing the test more than 9 days after the lab-based experiment (*N* = 2) or reporting that they had re-watched one of the TED-Ed videos since the lab-based experiment (*N* = 1). A linear mixed-effects model again revealed no significant main effect of condition on total follow-up quiz score (β = 0.29, *SE* = 0.20, 95% CI: [−0.089, 0.675], *p* = 0.14).

When gender and cognitive ability were included in the model as fixed effects, the model again revealed no significant main effect of condition on total follow-up quiz score (β = 0.26, *SE* = 0.19, 95% CI: [−0.114, 0.624], *p* = 0.18) and no main effect of gender on total quiz score (β = 0.05, *SE* = 0.21, 95% CI: [−0.458, 0.350], *p* = 0.80). However, there was a significant main effect of cognitive ability on total follow-up quiz score, with higher matrix reasoning scores predicting better quiz scores (β = 0.11, *SE* = 0.04, 95% CI: [0.032, 0.182], *p* < 0.01).

Finally, to determine if there was a difference in average response time to quiz questions based on incentive condition, we compared mean response time to quiz questions for participants in the in-lab quiz [Monetary condition: *M* = 15.48, *SE* = 0.51; No Incentive condition: *M* = 14.22, *SE* = 0.54], as well as the follow-up quiz [Monetary condition: *M* = 13.16, *SE* = 0.87; No Incentive condition: *M* = 13.22, *SE* = 0.70]. Results of the linear mixed-effects model revealed no significant main effect of condition on average response time for either the in-lab quiz (β = −0.07, *SE* = 0.05, 95% CI: [−0.156, 0.163], *p* = 0.16) or the follow-up quiz (β = 0.05, *SE* = 0.06, 95% CI: [−0.065, 0.025], *p* = 0.40).

In order to estimate the likelihood that the observed null effect of incentive condition on quiz score reflected a true underlying null distribution, we used a region of practical equivalence (ROPE) approach, a form of Bayesian inference (Kruschke, 2011). We set a series of narrow ROPE intervals considered practically equivalent to zero (i.e., no difference in total quiz points between conditions): ± 3, ± 2, and ± 1 (referring to difference in quiz points between conditions). Results showed that the majority of posterior estimates fell within these narrow ROPE intervals considered practically equivalent to no effect. Specifically, 100% of the incentive condition parameter distribution fell between ± a 3 quiz point difference, 100% of the incentive condition parameter distribution fell between ± a 2 quiz point difference, and 83.4% of the incentive condition parameter distribution fell between ± 1 quiz point (see Rodman et al., 2020 for more details on this approach). We interpret these results as strong evidence supporting a true underlying null effect of condition (Monetary vs. No Incentive) on total quiz score.

Overall, these results suggest that offering participants a monetary incentive for correct answers did not significantly improve their performance on a computer-based educational task. Moreover, these incentives had no significant effect on participants’ retention of learned information on an online multiple-choice content quiz taken one week later.

There are a few possible explanations for these results. First, offering 50 cents per question in the Monetary condition may not have provided *enough* incentive for participants to change their learning behavior (e.g., effort or attention devoted to the learning task). However, this amount of money is comparable to or greater than previous experimental research examining the effect of monetary incentives on physical and cognitive effort (e.g., Locke and Braver, 2008: 25 cents; Rodman et al., 2020: 10-75 cents; Small et al., 2005: 18 cents).

Second, it is possible that changing the “stakes” (by manipulating the presence or absence of a monetary incentive) in an online learning task simply has no effect on performance. Unlike other simple cognitive control experiments, the present test-taking scenario using educational video content provided a more naturalistic – and perhaps more engaging – learning environment. Thus, participants may simply be at ceiling in terms of their level of engagement or effort. In effect, they may have been driven by interest, the desire to learn, or the desire to obtain a high score, and thus exert a high amount of effort even in the absence of extrinsic incentives.

Lastly, it may be the case that incentives are only effective when students are able to *anchor* their value judgments (i.e., to directly compare one set of incentives to another). Given that the amount of effort humans exert seems to scale with the value of an incentive (Pessiglione et al., 2007; Schmidt et al., 2012; Rodman et al., 2020), perhaps participants will only put increased effort toward a “high-value” learning task when there is a baseline (that is, when they are able to compare it to a lower-value or no-value incentive). In Experiment 2, we sought to address this final possibility by manipulating the distribution of incentives *within subjects* in order to determine more conclusively whether or not monetary incentives can influence adults’ performance on online learning tasks.

## Experiment 2

Experiment 2 sought to replicate Experiment 1 in a within-subjects context. In particular, we directly tested the effect of learning from educational videos, where performance on some video content was incentivized monetarily, and performance on other content was not. A new set of participants were brought into the lab to watch the same four online educational videos (from Experiment 1) and answer the same 32 content-based quiz questions about what they learned. However, two of the videos (and corresponding sets of quiz questions) were assigned to be incentivized monetarily (*Monetary* videos) and two of the videos (and corresponding sets of quiz questions) were assigned to have no monetary incentive (*No Incentive* videos). Additionally, we again re-tested students one week after the initial learning episode to determine whether incentives differentially influence performance on a delayed learning task. If monetary incentives are in fact a strong motivator for students to direct their cognitive resources toward academic tasks – when anchored against no external incentives – then we expected participants to perform significantly better on Monetary video quiz questions compared to No Incentive video quiz questions, both immediately following the lesson and one week later.

### Method

#### Participants

50 healthy college-aged adults took part in Experiment 2 (32% male, 48% Caucasian, age range: 18-22, *M* = 19.86, *SD* = 1.09). The average score on the matrix reasoning subtest (our proxy for cognitive ability) for these participants was 24.26 (*SD* = 2.45), and, as in Experiment 1, the average level of background topic knowledge was low across participants (*M* = 2.06 (out of 5), *SD* = 0.63). Participants were recruited through Harvard University’s SONA Study Pool. Because this was a within-subjects study, we used less stringent exclusion criteria; participants were only excluded if they reported having a learning disability diagnosis or not being fluent in English. Two additional participants were tested but not included due to having a learning disability (*N* = 1) or already completing Experiment 1 (*N* = 1). Five additional participants were tested but excluded from all analyses for failing the manipulation check (described in the Procedure below).

Published data on laboratory experiments examining the effect of incentives on online learning was not available to inform *a priori* power analyses. Thus, we aimed to ensure that the sample size was sufficiently powered to detect medium-sized effects. A power analysis using the pwr2 package in R suggested that more than 33 within-subjects participants would be required for a medium effect size (Cohen’s *d* = 0.5) at *p* < 0.05 and 80% power. We chose to round this number up to 50 participants, given that 51 within-subjects participants would be required to detect a slightly smaller effect size (Cohen’s *d* = 0.4 at *p* < 0.05 and 80% power).

Participants provided informed consent for the present study, and all research procedures were approved by the Committee for Use of Human Subjects at Harvard University.

#### Materials

Materials for Experiment 2 were identical to Experiment 1 except in instructions (described in Procedure below).

#### Procedure

In Experiment 2, all participants watched the same four TED-Ed videos and answered the same 32 quiz questions as in Experiment 1. However, this time, two of the videos (and corresponding sets of quiz questions) were assigned to be incentivized monetarily (*Monetary* videos) and two of the videos (and corresponding sets of quiz questions) were assigned to have no monetary incentive (*No Incentive* videos). Which two videos were assigned to be Monetary or No Incentive varied across versions of the experiment (for a total of six different versions), and the order of videos/question sets was randomized across participants. Participants were randomly assigned to one of the six versions of the experiment.

The laboratory room set-up was identical to Experiment 1. At each in-lab session, participants arrived at a computer lab and were given a subject identification number. An average of 4.75 participants (*SD* = 5.15) took part in each session (range: 1-16). As in Experiment 1, participants were instructed to sit at an individual computer (separated from other computers by dividers), put headphones on, follow the instructions on their computer screen, and raise their hand if they had questions for the experimenter.

Participants first provided informed consent, answered demographic questions, and completed the matrix reasoning subtest (Wechsler, 2011). Next, participants received instructions for completing the in-lab learning task. As in Experiment 1, participants were told the following: “In this part of the study, you will watch four five-minute educational videos and answer questions about what you learned. You will complete a short quiz following each of the four videos.” Next, all participants also read the following instructions: “For some of the videos (**Money + Score)**, you will be told your score and receive bonus money based on how well you do (50 additional cents for each question you answer correctly). For the other videos (**Score Only**), you will be told your score but receive no bonus money. At the end of the task (32 questions total), you will see your final score for each video and be paid bonus money for each question you answered correctly for the Money + Score videos only (up to $8).”

Participants watched four TED-Ed videos and completed eight multiple-choice content questions immediately following each video (see Figure 1). Before each Monetary video, participants read the following: “This is a **Money + Score** video. After the video, you will complete a short quiz about what you learned. At the end of the study, you will receive your score as well as bonus money (50 cents) for each question you get correct.” Before each No Incentive video, participants instead saw the following: “This is a **Score Only** video. After the video, you will complete a short quiz about what you learned. At the end of the study, you will receive your score.”

To ensure that participants paid attention to the differing incentives, after each set of quiz questions, they were asked to report what kind of video they had just completed questions about (either “Money + Score” or “Score Only”). As mentioned about, five participants failed this manipulation check (each for only one out of four of the videos), and their data were excluded from all analyses.

At the end of the study, all participants were shown their total score for each video. All participants received standard compensation for participating in the lab study ($15 per hour or course credit). Participants also received 50 additional cents for every question they answered correctly about the Monetary videos only.

Approximately one week later (between 6-9 days following the in-lab experiment), participants completed an additional online “questionnaire.” The procedure for this follow-up quiz (and the quiz itself) was identical to Experiment 1.

#### Analytical Approach

Analyses examined the effect of incentive (Monetary vs. No Incentive) on total quiz score. As in Experiment 1, linear mixed-effects models were computed separately for the in-lab and online follow-up scores using the *lmer* function from the *lme4* (Bates et al., 2015) and *lmerTest* packages in R (Kuznetsova et al., 2017), with incentive (Monetary vs. No Incentive) as the key predictor of interest. Video type was included as a fixed effect (in order to account for any differences in difficulty across videos), and subject was included as a random effect (lme4 model formula: *Score* ∼ *Condition* + *Video* + (*1* | *Subject)*). As in Experiment 1, we also re-ran all models with gender (self-reported gender identity) and cognitive ability (as measured by the matrix reasoning subtest of the WASI-II) included as fixed effects (lme4 model formula: *Score* ∼ *Condition* + *Video* + *Gender* + *WASI* + (*1* | *Subject)*).

Moreover, we again ran analyses to determine if there was a difference in average response time to quiz questions based on incentive condition (Monetary vs. No Incentive). Linear mixed-effects models were again computed separately for the in-lab and online follow-up scores, with condition (Monetary vs. No Incentive) as the key predictor of interest. Video type was included as a fixed effect, and subject (i.e., participant) was included as a random effect (lme4 model formula: *Response Time* ∼ *Condition* + *Video* + (*1* | *Subject)*).

#### Results and Discussion

**FIGURE 3:**
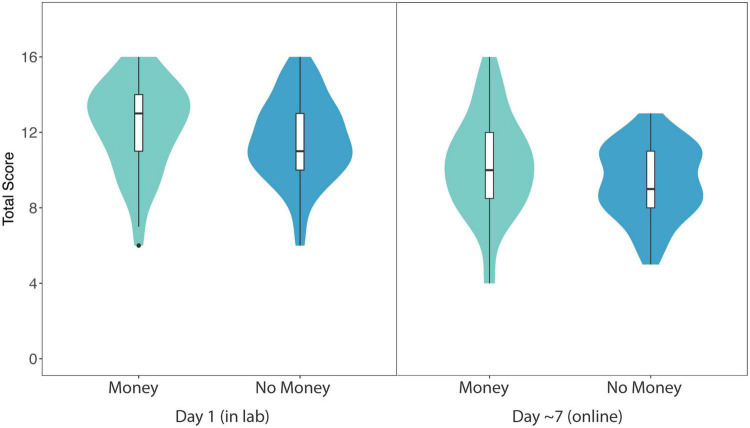
Violin plots showing probability density of total score data (within-subjects) for Monetary and No Incentive videos in Experiment 2 (in lab and online follow-up). Maximum score was 16 points (8 questions per video). Horizontal lines show median average score, surrounded by boxes indicating the interquartile range. Results revealed a significant effect of incentive (Monetary compared to No Incentive) on total score in both the lab experiment and the online follow-up.

Figure 3 displays total in-lab quiz scores (out of 16) for participants on Monetary videos (*M* = 12.32, *SE* = 0.33) and No Incentive videos (*M* = 11.60, *SE* = 0.31). Our primary linear mixed-effects model revealed a significant main effect of incentive (Monetary vs. No Incentive) on total quiz score (β = 0.36, *SE* = 0.18, 95% CI: [0.012, 0.708], *p* = *0.046*). Thus, providing a monetary incentive improved participants’ online learning performance by approximately one-third of a quiz question, on average, compared to the No Incentive condition.

When gender and cognitive ability were included as fixed effects, the model again revealed a significant main effect of incentive (Monetary vs. No Incentive) on total quiz score (β = 0.36, *SE* = 0.18, 95% CI: [0.012, 0.708], *p* = 0.046). There was no main effect of gender (β = 0.03, *SE* = 0.25, 95% CI: [−0.450, 0.506], *p* = 0.91) or cognitive ability (β = 0.01, *SE* = 0.05, 95% CI: [−0.104, 0.080], *p* = 0.81) on total quiz score.

To examine the influence of incentives on delayed quiz performance, we compared mean total follow-up quiz scores (out of 16) for participants on Monetary videos (*M* = 10.23, *SE* = 0.38) and No Incentive videos (*M* = 9.38, *SE* = 0.28) one week later (see Figure 3). Again, a linear mixed-effects model revealed a significant main effect of incentive (Monetary vs. No Incentive) on total follow-up quiz score (β = 0.35, *SE* = 0.16, 95% CI: [0.033, 0.673], *p* = 0.03). As before, providing an initial monetary incentive improved participants’ online learning performance in a delayed test of learning by approximately one-third of a quiz question, on average, compared to the No Incentive condition.

When gender and cognitive were included in the model as fixed effects, the model again revealed a significant main effect of incentive (Monetary vs. No Incentive) on total follow-up quiz score (β = 0.35, *SE* = 0.16, 95% CI: [0.033, 0.673], *p* = 0.03). However, there was no main effect of gender (β = 0.03, *SE* = 0.29, 95% CI: [−0.588, 0.529], *p* = 0.92) or cognitive ability (β = 0.06, *SE* = 0.05, 95% CI: [−0.049, 0.162], *p* = 0.31) on total follow-up quiz score.

Finally, to determine if there was a difference in average response time to quiz questions based on incentive condition, we compared mean response time to quiz questions for participants in the in-lab quiz [Monetary condition: *M* = 16.26, *SE* = 0.73; No Incentive condition: *M* = 13.82, *SE* = 0.40], as well as the follow-up quiz [Monetary condition: *M* = 11.59, *SE* = 0.63; No Incentive condition: *M* = 12.76, *SE* = 1.32]. Our linear mixed-effects model revealed a significant main effect of condition on average response time for the in-lab quiz (β = − 0.10, *SE* = 0.04, 95% CI: [−0.179, −0.014], *p* = 0.02), but no significant main effect of condition on average response time for the follow-up quiz (β = 0.07, *SE* = 0.04, 95% CI: [−0.018, 0.153], *p* = 0.12).

These results suggest that offering participants a monetary incentive for correct answers significantly improved their performance on online learning questions, relative to their performance on questions for which they had been offered no monetary incentive. In other words, when participants were given the opportunity to *anchor* their interpretation of the incentive and compare it to a lower-stakes situation (i.e., receiving 50 cents per question relative to no cents per question), the monetary incentive had a significant influence on their online learning performance. Moreover, the fact that participants spent significantly longer responding to quiz questions that were monetarily incentivized suggests that they may have put more *effort* into answering those questions. In the one-week follow-up, when there was no longer any additional incentive, participants showed no significant difference in response time across question type. However, *learning* from the incentivized videos seemed to persist over time; in the follow-up quiz, participants performed significantly better on new questions testing content from incentivized videos compared to questions testing content from videos that had not been incentivized.

## General Discussion

In two experiments, we tested whether changing the “stakes” in a computer-based educational task – by manipulating the presence or absence of a monetary incentive – can influence performance. In Experiment 1, we examined the online learning performance of a group of participants who received a monetary incentive for their scores compared to a group who received no additional incentive (beyond personal achievement). We found no significant difference in performance or effort (measured by average question response time) between these two groups, suggesting that there is no inherent effect of monetary incentives on online learning performance. In Experiment 2, however, we tested whether there was an influence of incentives on online learning performance if participants were able to directly compare types of incentive (monetary vs. no external incentive). In a within-subjects design, we found that participants performed significantly better on online learning questions when they were incentivized monetarily, compared to questions where they received no external incentives. These participants also had a significantly longer response time to incentivized questions, suggesting that participants put forth additional cognitive effort during the quiz phase when money was on the line. In an online follow-up, the learning effect persisted, despite the fact that monetary questions were no longer being incentivized (and no additional response time – or effort – was being put into answering those questions). Thus, the present results suggest that monetary incentives *can* drive cognitive effort (and subsequent learning) in an online learning context, but only when participants can directly compare the incentivized context against a context with no external incentive.

The fact that monetary incentives drive cognitive effort only when presented in direct comparison to a baseline level of no incentive aligns with the perspective that cognitive effort exertion tracks with amount of incentive. That is, participants have been shown to exert more cognitive effort when presented with high-incentive compared to low-incentive cues (Bijleveld et al., 2009; Capa et al., 2011; Schmidt et al., 2012). Anchoring value judgments may be particularly important given that “value” itself is subjective (see Davidow et al., 2018). That is, monetary incentives can mean different things to different people, and this may be particularly true when there is no alternative by which to compare an incentive.

An alternative explanation for our findings is that differential performance for Monetary and No Incentive video quiz questions in Experiment 2 may have revealed a version of the “undermining effect” (e.g., Braver et al., 2014). That is, perhaps the external incentive did not directly cause an *increase* in cognitive effort compared to baseline, but rather, participants may have *decreased* their motivation to perform well on the online learning task when the monetary incentive was “taken away.” However, the increase in online learning performance for monetary quiz questions compared to no incentive questions persisted at the one-week follow-up, when no external incentive was being offered. At the same time, while participants showed a longer response time to monetary quiz questions at the initial test (indexing increased effort toward answering incentivized questions), there was no significant difference in response time to monetary (compared to no incentive) questions at the one-week follow-up, in the absence of an external incentive. Thus, participants were not simply less motivated to perform once the incentive had been removed, but rather, they seemed to have actually *learned* the content better when an incentive had previously been offered (likely due to an initial increase in cognitive effort). This persisting learning effect aligns with previous research showing that reward can enhance long-term memory (e.g., Murayama and Kitagami, 2014).

Regardless of the precise underlying mechanism, it does seem to be the case that monetary incentives were only effective at increasing online learning performance when participants were able to anchor their value judgments (i.e., to directly compare one set of incentives to another). What might this mean in an educational context? Perhaps teachers and educational developers can use incentives to help students prioritize high- or low-value assignments or academic content, or perhaps students can utilize this practice in developing their own study techniques. The use of these kinds of incentives may also align with ideas surrounding the gamification of education, or the use of game-like features to increase engagement, particularly in online learning contexts (e.g., Landers and Callan, 2011; Muntean, 2011). Importantly, however, one gamification study found that students in a gamified course had significantly lower self-rated intrinsic motivation and course satisfaction over time compared to students in a non-gamified course (Hanus and Fox, 2015). Yet other research has shown that external rewards in an educational game (in particular, “badges” to indicate achievements) do *not* undermine students’ motivation, and in fact, may have a positive influence on their conceptual understanding and performance (Filsecker and Hickey, 2014). Given the mixed results of external rewards in the gamification literature (Kyewski and Krämer, 2018), future research should continue to examine how the use of monetary incentives may alter student behaviors over time, throughout the length of a course.

Importantly, we do not aim to suggest that students are not *also* driven by such factors as personal interest, the desire to learn, or even the desire to obtain a high score. Even in the within-subjects experiment, the effect of monetary incentives on performance was relatively small. However, the present research does suggest that the use of external incentives may push students to exert additional cognitive resources toward learning, studying, or performance in certain contexts. Additionally, in the present study, we focused on *monetary incentives* as a test case for external incentives, based on decades of research in cognitive psychology using money as a representation of “stakes” (Davidow et al., 2018). However, there are a variety of other educationally-relevant external incentives (such as student ranking information, private or public praise, awards, and letters of recommendation). Future research should work to better understand differences in the use of external incentives and their influence on online learning performance.

Additionally, while the present study aimed to test the influence of monetary incentives on a computer-based online learning task in particular, future studies should clarify the extent to which these findings generalize to in-person learning. Previous studies have shown the difficulty in equating online learning with in-person learning; for example, some research has found online learning to be more effective than in-person learning (e.g., Schoenfeld-Tacher et al., 2001), while other research suggests that learners (especially young children) tend to learn less from screen-based compared to face-to-face instruction (see Strouse and Samson (2021) for a recent meta-analysis of this “video deficit”). Given possible differences in intrinsic motivation across online and in-person formats (Alfonsson et al., 2017), future work should compare the influence of monetary incentives in online and classroom-based learning to inform the generalizability of these results to different learning contexts.

Based on the presents results, we tentatively suggest that monetary incentives may increase performance in computer-based online learning tasks, at least when participants are able to anchor the “stakes” of an incentive compared to no external incentive. Before making any sweeping recommendations for the education system, future research should further examine (1) the extent to which this effect persists over time in real academic settings, (2) the effect of other educationally-relevant extrinsic incentives on the learning process, and (3) any potential negative effects of incentives on long-term intrinsic motivation. Nevertheless, we view the present research as a first step toward integrating research from the fields of education, economics, and cognitive psychology in order to highlight potential benefits of external incentives for promoting cognitive effort and learning in an online educational context. Ultimately, we hope that instructors and educational developers can apply research-based principles of motivation and behavior to improve students’ engagement and learning, particularly in online course contexts.

## Data Availability Statement

The datasets presented in this study can be found online at: https://osf.io/wcbsn/.

## Ethics Statement

The studies involving human participants were reviewed and approved by Harvard University Area Institutional Review Board. The participants provided their written informed consent to participate in this study.

## Author Contributions

JS and LS contributed to the conception and design of the study. JS conducted the study, performed statistical analyses, and wrote the first draft of the manuscript. Both authors contributed to manuscript revision and approved the submitted version.

## Conflict of Interest

The authors declare that the research was conducted in the absence of any commercial or financial relationships that could be construed as a potential conflict of interest.

## Publisher’s Note

All claims expressed in this article are solely those of the authors and do not necessarily represent those of their affiliated organizations, or those of the publisher, the editors and the reviewers. Any product that may be evaluated in this article, or claim that may be made by its manufacturer, is not guaranteed or endorsed by the publisher.
