# Supplementary material for: Raising the Stakes for Online Learning: Monetary Incentives Increase Performance in a Computer-Based Learning Task Under Certain Conditions
Source: Front Psychol. 2022 May 5;13:780301. doi: 10.3389/fpsyg.2022.780301 (PMC9119014; doi:10.3389/fpsyg.2022.780301)
Supplement: Supplementary file 1 [file Data_Sheet_1.pdf]

Supporting Information

Additional method and results for Experiment 1.

In Experiment 1, 53 additional participants were tested in a "Social Ranking" condition, not reported in the body of the paper. The methods and procedure for participants in the Social Ranking condition were identical to those in the Monetary and No Incentive condition, except participants were given the following instructions: "You will also receive information about how well you do on this online learning task compared to your peers. At the end of the task (32 questions total), you will see your final score compared to the average score of other participants." At the end of the task, participants were shown their own final score, as well as the average score of participants who had completed the research study to date.

First, we found no significant difference in raw scores on the matrix reasoning subtest between participants in the Social Ranking condition ($M = 24.08$, $SE = 0.39$) and No Incentive condition ($M = 23.92$, $SE = 0.32$), $t(104) = 0.30$, $p = 0.77$, $d = 0.06$. We also found no significant difference in raw scores on the matrix reasoning subtest between participants in the Social Ranking condition ($M = 24.08$, $SE = 0.39$) and Monetary condition ($M = 24.28$, $SE = 0.36$), $t(104) = 0.39$, $p = 0.70$, $d = 0.08$. This suggests that participants did not differ across conditions in terms of general cognitive ability.

Additionally, mean total in-lab quiz scores (out of 32) were comparable for participants in the Social Ranking condition ($M = 22.85$, $SE = 0.50$) compared to participants in the Monetary condition ($M = 23.70$, $SE = 0.50$) and No Incentive condition ($M = 23.66$, $SE = 0.61$). To estimate the effect of condition (Social Ranking, Monetary, or No Incentive) on total quiz score, we re-ran our primary linear mixed-effects model (with condition as our key predictor of interest, video type as a fixed effect, and subject as a random effect) using the *lmer* function in R (Bates

et al., 2015; Kuznetsova, Brockhoff, & Christensen, 2017). The model revealed no significant difference in performance between the Monetary and No Incentive conditions ($\beta = 0.01.$, SE = 0.19, 95% CI: [-0.362, 0.381], $p = .96$), and no significant difference in performance between the Social Ranking and No Incentive conditions ($\beta = -0.20.$, SE = 0.19, 95% CI: [-0.575, 0.169], p = 0.28). To compare all three conditions, we ran post-hoc comparisons using the phia package in R (De Rosario-Martinez, 2015), using Holm's method to correct for multiple comparisons. The model again revealed no significant main effect of condition on total quiz score (χ^2 (2) = 1.59, p = 0.45).

Mean follow-up quiz scores (out of 32) obtained one week later were also comparable for participants in the Social Ranking condition (M = 18.55, SE = 0.58) compared to the Monetary condition (M = 19.84, SE = .50) and No Incentive condition (M = 18.56, SE = 0.61). Note that two additional participants from the Social Ranking condition did not complete the follow-up, and we excluded scores of two additional participants for completing the test more than 9 days after the lab-based experiment (N = 2). We again ran a linear mixed-effects model to estimate the effect of condition (Social Ranking, Monetary, and No Incentive) on total follow-up quiz score, with condition as our key predictor of interest, video type as a fixed effect, and subject as a random effect using the *lmer* function in R (Bates et al., 2015; Kuznetsova, Brockhoff, & Christensen, 2017). The model revealed no significant difference in performance between the Monetary and No Incentive conditions ($\beta = 0.29.$, SE = 0.20, 95% CI: [-0.091, 0.678], $p = .14$), and no significant difference in performance between the Social Ranking and No Incentive conditions ($\beta = 0.04.$, SE = 0.20, 95% CI: [-0.345, 0.428], p = 0.83). To compare all three conditions, we ran post-hoc comparisons using the phia package in R (De Rosario-Martinez,

2015), using Holm's method to correct for multiple comparisons. The model again revealed no significant main effect of condition on total quiz score (χ^2 (2) = 2.59, p = 0.27).

In order to estimate the likelihood that the observed null effect of incentive condition on quiz score reflected a true underlying null distribution, we used a region of practical equivalence (ROPE) approach, a form of Bayesian inference (Kruschke, 2011). We set a series of narrow ROPE intervals considered practically equivalent to zero (i.e., no difference in total quiz points between conditions) to compare performance between conditions (Monetary vs. No Incentive and Social Ranking vs. No Incentive). The intervals chosen were again +/- 3, +/- 2, and +/- 1 (referring to difference in quiz points between conditions). Results showed that the majority of posterior estimates fell within these narrow ROPE intervals considered practically equivalent to no effect. For the Monetary vs. No Incentive comparison, 100% of the incentive condition parameter distribution fell between +/- a 3 quiz point difference, 100% of the incentive condition parameter distribution fell between +/- a 2 quiz point difference, and 84.99% of the incentive condition parameter distribution fell between +/- 1 quiz point. For the Social Ranking vs. No Incentive comparison, 100% of the incentive condition parameter distribution fell between +/- a 3 quiz point difference, 97.05% of the incentive condition parameter distribution fell between +/- a 2 quiz point difference, and 61.14% of the incentive condition parameter distribution fell between +/- 1 quiz point. We interpret these results as strong evidence supporting a true underlying null effect of condition (Social Ranking or Monetary) on total quiz score.

Thus, including the third condition revealed a pattern of results that was identical to the results reported in Experiment 1. Regardless of how we analyze the data, we found no significant influence of additional external incentive (either Monetary or Social Ranking) on participants'

total in-lab quiz scores or follow-up quiz scores in a context where there was no direct comparison of incentives among participants.
